# Circulating tumour DNA alterations: emerging biomarker in head and neck squamous cell carcinoma

**DOI:** 10.1186/s12929-023-00953-z

**Published:** 2023-08-09

**Authors:** Xiaomin Huang, Pascal H. G. Duijf, Sharath Sriram, Ganganath Perera, Sarju Vasani, Lizbeth Kenny, Paul Leo, Chamindie Punyadeera

**Affiliations:** 1 Saliva and Liquid Biopsy Translational Laboratory, Griffith Institute for Drug Discovery (GRIDD), School of Environment and Science, Griffith University, QLD Brisbane, Australia; 2 School of Biomedical Sciences, Faculty of Health, Queensland University of Technology, Brisbane, QLD Australia; 3 Centre for Genomics and Personalised Health, Queensland University of Technology, Brisbane, QLD Australia; 4 Centre for Data Science, Queensland University of Technology, Brisbane, QLD Australia; 5 Institute of Clinical Medicine, Faculty of Medicine, University of Oslo, Oslo, Norway; 6 Department of Medical Genetics, Oslo University Hospital, Oslo, Norway; 7 University Queensland Diamantina Institute, The University of Queensland, Translational Research Institute, Brisbane, QLD Australia; 8 Functional Materials and Microsystems Research Group and the Micro Nano Research Facility, RMIT University, Melbourne, Australia; 9 Department of Otolaryngology, Royal Brisbane Women’s Hospital, Brisbane, QLD Australia; 10 The School of Medicine, University of Queensland, Royal Brisbane and Women’s Hospital, Brisbane, QLD Australia; 11 Australian Translational Genomics Centre, Brisbane, QLD Australia; 12 Menzies Health Institute Queensland (MIHQ), Griffith University, Gold coast, QLD Australia

**Keywords:** Head and neck cancer, Circulating tumour DNA, Tumour DNA, DNA alterations, Biomarkers, Liquid biopsy, Precision medicine

## Abstract

Head and Neck cancers (HNC) are a heterogeneous group of upper aero-digestive tract cancer and account for 931,922 new cases and 467,125 deaths worldwide. About 90% of these cancers are of squamous cell origin (HNSCC). HNSCC is associated with excessive tobacco and alcohol consumption and infection with oncogenic viruses. Genotyping tumour tissue to guide clinical decision-making is becoming common practice in modern oncology, but in the management of patients with HNSCC, cytopathology or histopathology of tumour tissue remains the mainstream for diagnosis and treatment planning. Due to tumour heterogeneity and the lack of access to tumour due to its anatomical location, alternative methods to evaluate tumour activities are urgently needed. Liquid biopsy approaches can overcome issues such as tumour heterogeneity, which is associated with the analysis of small tissue biopsy. In addition, liquid biopsy offers repeat biopsy sampling, even for patients with tumours with access limitations. Liquid biopsy refers to biomarkers found in body fluids, traditionally blood, that can be sampled to provide clinically valuable information on both the patient and their underlying malignancy. To date, the majority of liquid biopsy research has focused on blood-based biomarkers, such as circulating tumour DNA (ctDNA), circulating tumour cells (CTCs), and circulating microRNA. In this review, we will focus on ctDNA as a biomarker in HNSCC because of its robustness, its presence in many body fluids, adaptability to existing clinical laboratory-based technology platforms, and ease of collection and transportation. We will discuss mechanisms of ctDNA release into circulation, technological advances in the analysis of ctDNA, ctDNA as a biomarker in HNSCC management, and some of the challenges associated with translating ctDNA into clinical and future perspectives. ctDNA provides a minimally invasive method for HNSCC prognosis and disease surveillance and will pave the way in the future for personalized medicine, thereby significantly improving outcomes and reducing healthcare costs.

## Introduction

Head and neck cancers (HNCs) are the 7th most common cancer in the world, with 931,922 new cases and 467,125 deaths in 2020 [142]. The Organization Global Cancer Observatory estimates that the number of HNSCC patients will rise by 30% in 2030 [142]. Over 90% of head and neck malignancies are squamous cell carcinomas (SCCs). HNSCCs generally originate from the squamous cells lining the mucosal surfaces inside the head and neck region. They can be categorized by anatomical location: oral cavity, pharynx (nasopharynx, oropharynx, hypopharynx), larynx, paranasal sinuses, nasal cavity, and salivary gland cancer [24].

The International Agency for Research on Cancer (part of the World Health Organization) has identified diverse risk factors that contribute to the development of HNSCC. Excessive consumption of alcohol and tobacco use are the two major risk factors for the development of HNSCC. It is estimated that at least 75% of HNSCCs are caused by tobacco smoking and alcohol consumption [10, 11, 56]. Heavy users of both cigarettes and alcohol have a 35-fold higher risk of developing the disease [10]. High-risk human papillomavirus (HPV) [2, 19] and Epstein-Barr virus (EBV) [166] infections are also important risk factors for the development of oropharyngeal cancers and nasopharyngeal carcinomas (NPC) respectively. Certain types of viruses are common in certain communities. For example, in the Chinese population, especially the Cantonese living in Southern China, they have a higher incidence of EBV associated NPC [167]. Betel quid products are linked to a high incidence rate of oral cavity cancer in China and India [47]. Abnormal eating habits such as intake of preserved or salted food and diet lacking in vegetables [39], have been shown to increase morbidity. In low- and middle-income countries, occupational exposure to carcinogenic air pollutants is closely linked to the development of HNSCC [89]. Gender also matters, as compared to women, men are at 2 to fourfold higher risk of developing HNSCC [64]. Genetic factors can also predispose to the development of HNSCC [35]. It has been demonstrated that people with Fanconi anemia (a rare inherited genetic disease) have a 500–700-fold higher risk of developing HNSCC [7, 157]. In addition, people with poor oral health are also at a higher risk of developing HNSCC [50, 141]. Also, people who have not had the fortune of being vaccinated with HPV vaccination (Gardasil^®^) are at risk of developing HPV associated oropharyngeal squamous cell carcinoma (OPSCC) [1]. It is also known that patients with HPV-positive OPSCC have a more favorable prognosis than HPV-negative OPSCC [64].

### Current diagnostics and treatment strategies for managing patients with HNSCC

Current diagnostic methods for HNSCC include physical examination, endoscopy, imaging studies, biopsy and tumour biomarker testing [120]. Tissue biopsy either by resection or fine-needle aspiration (FNA) is invasive, and in some cases, it is difficult to access the tumour due to its anatomical location. Also, FNA biopsy results in a small of amount of tumour tissue that is available for both histologic diagnosis/subtyping and genetic testing for most advanced stage cancer patients, and in most instances, the tissue often becomes insufficient for genomic analysis after initial histology diagnosis. In addition, inter- and intra-tumoral heterogeneity may also limit the tumour tissue-based genotyping, and this issue amplifies when determining mechanisms for treatment resistance [130]. Therefore, alternative diagnostic methods are warranted.

Currently available treatments for patients with HNSCC include surgery, radiotherapy, chemotherapy, targeted therapy and immunotherapy [110]. Treatment decision making is currently based on the tumour-node-metastasis (TNM) stage, tumour p16 status, anatomic site, performance status (a scoring system that quantifies cancer patients' activity of daily life and overall well-being and activities of daily life) and patient preferences. For example, for patients with locoregionally advanced oral cavity cancer (OC), the first line of treatment is surgery, whereas chemoradiotherapy (CRT) is more commonly used for oropharyngeal (OPC) and laryngeal cancers (LC) [64]. Postoperative radiation and postoperative chemotherapy are usually applied to patients with pathological risk factors of developing recurrence and metastasis [25]. Immunotherapies are currently approved to treat HNSCC patients with recurrence or metastasis, such as Pembrolizumab (Keytruda^®^) and Nivolumab (Opdivo^®^), they are recommended in the National Comprehensive Cancer Network (NCCN) guideline.

### The need for biomarkers to triage patients with head and neck cancers

In recent decades, new cases of OPSCC are increasing globally due to increasing rates of HPV infections. OPSCC has now surpassed cervical cancer to become the number one cause of HPV-related cancer in the United States and in Australia [24, 155]. There are differences in terms of molecular mechanisms and oncogenic process between HPV-positive OPSCCs and HPV-negative HNSCCs. A better prognosis can be seen in HPV-positive OPSCC [77]. One of the ways to early detect OPSCC is to initiate a screening program targeting individuals within the community who are at a higher risk. However, this approach has not been applied in many countries where OPSCC disease burden is high due to our inability to detect occult OPSCC. This perspective changed in 2020, with the detection of 2 mm occult OPSCC at the back of the throat of an asymptomatic in a healthy individual, a world first, using serial saliva testing for HPV [146].

Before biomarkers can be integrated into a clinical workflow, they will have to undergo five phases of development (preclinical exploratory work, clinical assay development for clinical disease, retrospective longitudinal repository studies, prospective screening studies and cancer control studies [114]) to prove their clinical utility in terms of sensitivity and specificity. Biomarkers that can be sampled using non-invasive methods (saliva and urine) will be a game changer, especially managing patients living in rural and remote communities. During the COVID-19 pandemic, the urgency for rapid, non-invasive, remote testing has come to the forefront, in which salivary diagnostics is showing promise as an alternative diagnostic medium to blood and tumour tissue testing [68]. Salivary diagnostics is still in a research phase but is expected to transform healthcare practice because of its ease of collection and the ability to be done at the conform of one’s home [160].

### The application of liquid biopsy to head and neck cancers

The use of precision, targeted genomic therapies in HNSCC lagged behind many other cancer types, leading to poor survival outcomes. As an example, oncogenic *PI3KCA* mutations are commonly found in HNSCCs [90], and patients with this mutation are most likely to benefit from PI3K pathway inhibitor treatment [46]. However, mutation analysis of the tumour tissue is not routinely done for HNSCC. This would mean that those patients with *PI3KCA* mutations would not benefit PI3K pathway inhibitor. Given the drawback of using tumour tissue to diagnose and predict treatment response in HNSCC patients, alternative methods are urgently needed to better manage patients with HNSCC [130]. Liquid biopsy, the use of cancer specific biomarkers that are present in body fluids to evaluate tumour activities and to discern underlying disease pathogenesis, is an emerging field in oncology. Some of the biomarkers used include CTC, ctDNA/RNA and exosome, to name a few. The ease of sampling and the ability to collect multiple samples “in real-time” from a cancer patient makes liquid biopsy as an alternative tool to managing HNC patients.

### Origin of cell-free DNA and circulating tumour DNA

Mandel and Metais in 1948 detected for the first time cell-free DNA (cfDNA), which is now referred to as short fragments of nuclear acids in circulation [91]. cfDNA is released from both normal and tumour cells into circulation through cellular apoptosis and necrosis [62], having a half-life of 10–15 min. cfDNA is degraded by blood nucleases, and/or eliminated by macrophages in kidney, liver and spleen [148, 159].

**Fig. 1:**
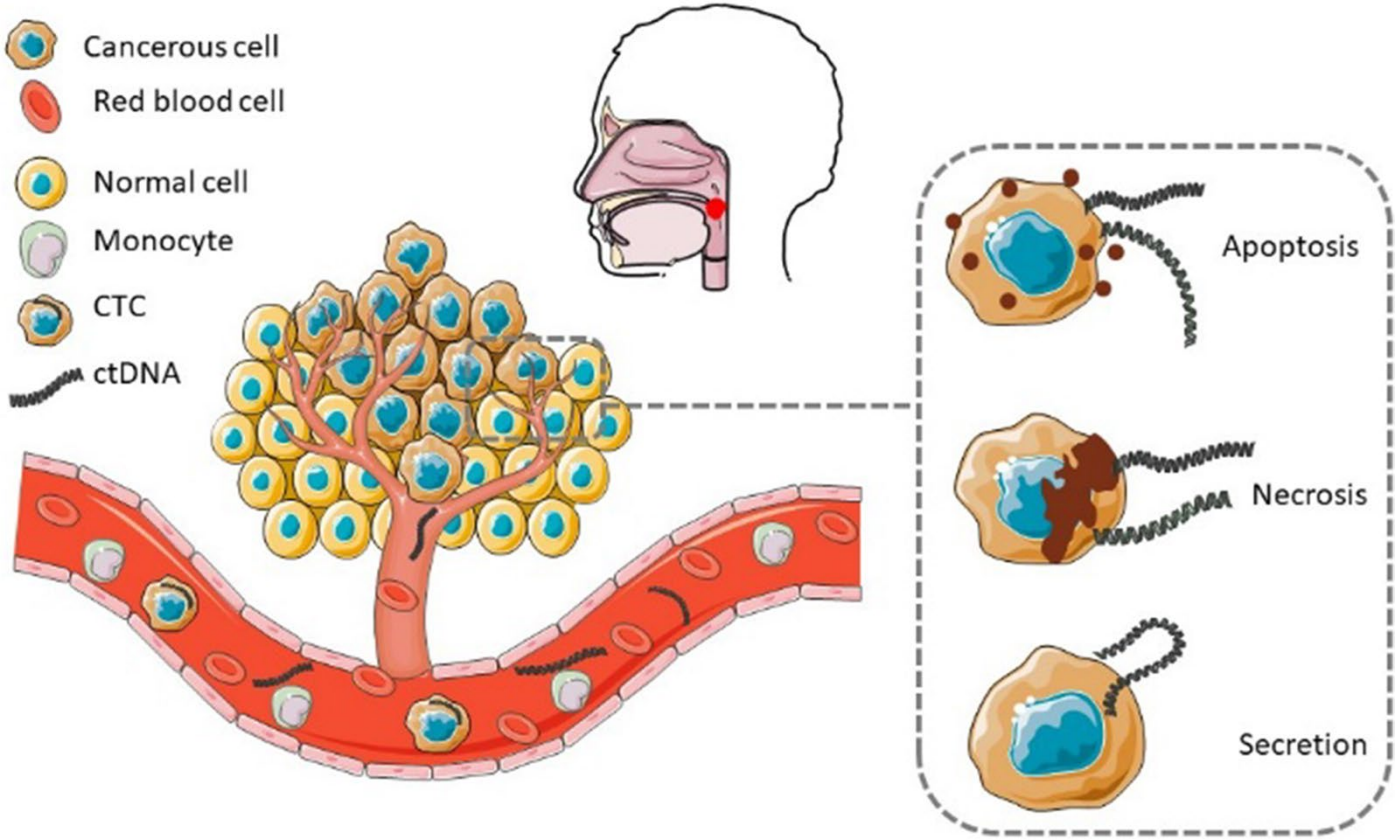
ctDNA originates from the tumour and is released into circulation

Circulating tumour (ctDNA) is derived from tumour and is part of the total cfDNA pool, representing only small fraction of cfDNA. It ranges from 0.01% to 90% [36], but is usually less than 1% of the total cfDNA. ctDNA is released either through passive (apoptosis and necrosis) or active secretion (Fig. 1). ctDNA fragments that are released into circulation due to apoptosis are of 160 bp–180 bp, whilst the ctDNA fragments that are actively secreted into circulation are of 150 bp–250 bp. In contrast, ctDNA released through necrosis are much larger, ranging from 320 bp to more than 1000 bp [36]. In addition, the lysis of CTCs also thought to contribute to the volume of ctDNA detected in circulation, although the exact mechanism is still not well understood [113]. Studies have found high molecular weight DNA fragments associated with ctDNA through electrophoresis techniques, and this is released into circulation due to cell lysis [48]. Sutton et al. [145]*,* revealed that M2-like tumour-associate macrophages (TAMs) can regulate CTCs metastasis by breaking down the basement membrane, promoting angiogenesis and protecting tumour cells from anti-tumour immunization [145]. TAMs can lyse CTCs through phagocytosis and release DNA into circulation [138]. The amount of ctDNA in circulation is influenced by the type of cancer, stage of the tumour, cancer burden, cellular turnover, and therapy response [32]. Muhanna et al. found that the volume of tumour necrosis was positively correlated with plasma ctDNA in a preclinical rabbit model of HNSCC [101].

### Overview of the tumour mutational landscape in head and neck squamous cell carcinoma

**Fig. 2:**
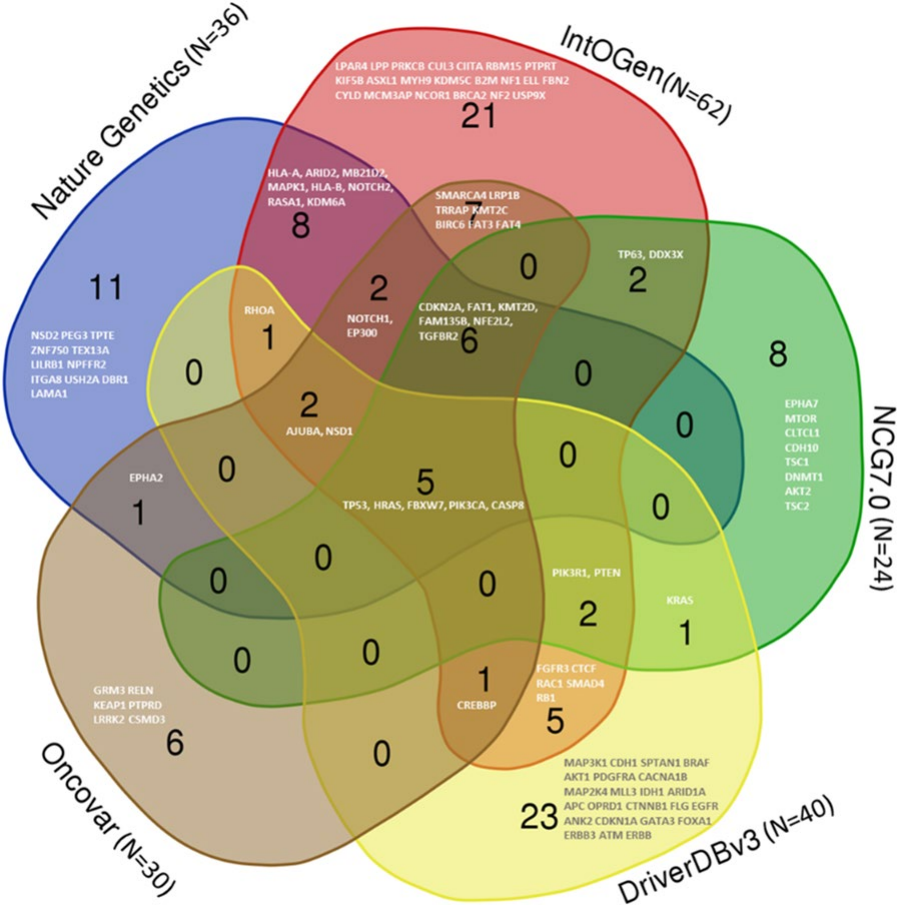
A Venn graph illustrating the numbers of head and neck cancer driver genes in five databases. 5 genes are included in all the databases, 8 genes are included in four databases, 6 genes are included in three databases, and 24 genes are included in two databases

The mutational profiles of HNSCC have been reported by The Cancer Genome Atlas (TCGA) and cBioPortal databases. In general, the majority of HNSCCs present loss of function in tumour suppressor genes [139], and this is also common in genes regulating key cell cycle and cell differentiation pathway. By definition, a driver gene is defined as “a gene whose mutations accelerate net cell growth” [152], but there is no gold standard to identify driver genes. So far, they are mostly defined by computational algorithms that model the genes tumour specific rates compared to its hypothetical background. There are several databases for cancer driver genes/driver mutations, including, Integrative OncoGenomics (N = 691 for HNC) [94], Network of cancer genes and healthy drivers (NCG 7.0, N = 1002 for HNC) [122], Oncovar (N = 2798 for HNC data available in TCGA) [161]*,* DriverDBv3 (N = 2798) [88]*.* Also, Dietlein et al*.,*’s publication in Nat Genet (N = 425 for HNC) [33] summarized different cancer drivers in over 28 cancer types*.* Notably, the mutational profiles between HPV-negative HNC and HPV-positive HNC are different [134], they are likely to have different driver genes because of their biological differences. Figure 2 summarizes HNC driver genes that have been reported in the five databases mentioned above. We are reporting the diver genes that have been reported in more than two databases, along with their roles in HNSCC.

### Driver gene mutations in head and neck squamous cell carcinoma

The most common mutation in HPV-negative HNSCC is *TP53*, whose mutations are in 73–100% of HPV-negative HNSCC cases [152]. *TP53* is a tumour suppressor gene that functions as a gatekeeper for cell growth and division [79]. This involves, arresting cells in cell cycle, initiating apoptosis or senescence when there are errors in cellular DNA synthesis and replication. However, *TP53* mutation is rarely seen in HPV-positive HNSCC, this may be because of the HPV E6 viral protein initiating the degradation of *TP53* [5]. The presence of *TP53* mutation can be regarded as an early event during tumourigenesis in HNSCC [124]. HNSCC patients who have a *TP53* mutation usually respond poorly to cisplatin-fluorouracil neoadjuvant chemotherapy [14], leading to local recurrence after radiation therapy [44]. In addition, *TP63* (tumour protein 63) encodes a member of the p53 family of transcription factors. Recurrent focal amplification for 3q26/28 involving the *TP63* locus occurs in 15% of HNSCC [16].

Cyclin D1 (*CCND1*) and cyclin dependent kinase inhibitor 2A *(CDKN2A)* are two genes involved in cell cycle and DNA repair pathways. Amplification of *CCND1* and deletion of *CDKN2A* occur in 94% of oral squamous cell carcinoma (OSCC) [76] and structural alterations (homozygous deletion, intra and inter chromosomal fusions) appear to be prominent in *CDKN2A* [16]. In addition, studies have shown a mutation (8–12%) and a homozygous deletion in Protocadherin *FAT1* (FAT atypical cadherin 1) (6%) in HNSCC [139]. Moreover, functional loss of *FAT1* either by mutation or homozygous deletion can activate Wnt signaling pathway to promote tumorigenesis [100].

Epidermal growth factor receptor *(EGFR)* is a receptor tyrosine kinase (RTK) that is frequently altered in HNSCC [98] and stimulation of *EGFR* or other RTKs can active the PI3K/Akt pathway. More than 10% HNSCC patients have amplifications on *EGFR* of chromosome 7 [124, 147]. Grandis et al. observed that *EGFR* copy number variations (CNV) is linked to poor prognosis in HNSCC [49].

Ajuba LIM Protein *(AJUBA)****,*** a gene in WNT/β-catenin signaling pathway, is found to inactivate mutations in HPV-negative HNSCC [6]. It can negatively regulate the NOTCH1/CTNNB1 signaling pathway [8].

Family with sequence similarity 135, member (B*FAM135B)* is a cancer-related gene on chromosome 8q. It has been shown to increase progression of esophageal squamous cell carcinoma (ESCC), and mutation of *FAM135B* in ESCC corelated to poor prognosis [137]. Its mutation rate in HNSCC is relatively high, mostly missense mutations in > 10% of patients [33], but its exact role in HNSCC remains to be explored.

Ras homolog family member A *(RHOA)* encodes a small GTPase in the Rho family, regulating cell motility and tissue development [61]. *RHOA* mutations in gastric cancers [153] can induce cell proliferation [60], its role in HNSCC remains yet to be explored.

Nuclear receptor-binding SET domain protein 1 (*NSD1*) encodes a protein containing a SET domain. Truncating mutations, novel focal deletions (includes homozygous deletions and inframe deletions), missense point mutations and inactivating mutations are found in *NSD1* in HNSCC [16, 111]. In laryngeal cancer, inactivating mutations in *NSD1* are seen as a favorable prognostic biomarker [116].

Nuclear Factor Erythroid 2-Related Factor 2 (*NFE2L2*) acts as an oxidative stress factor regulating antioxidant and stress-responsive genes**.** It only mutates in HPV-negative HNSCC, and heavily related to smoking [8].

Caspase-8 (*CASP8*) is located on chromosome 2 and is involved in cell death through the death receptor pathway. Knockdown of *CASP8* makes HNSCCs susceptible to necroptosis [154]. Li et al. illustrated a six-nucleotide deletion variant (− 652 6N del) in the promoter region of *CASP8*, inversely contributing to the risk of HNSCC development [80]. A lower *CASP8* mutation frequency is associated with lower aggressiveness in HNSCC. In addition, *CASP8* mutations are found in 10% OSCC tumours [117].

In HPV-positive HNSCC, PI3K/Akt signaling pathway is the most mutated signaling pathway [90] and has shown to correlate with genomic instability. The PI3K/Akt pathway is involved in cell proliferation, survival and morphology [109]. Concurrent mutations of multiple PI3K pathway genes have been shown in patients with advanced-stage HNC [90]. PI3K/Akt mutations are associated with the anatomical site where the tumour originates from, particularly in anatomical locations such as the larynx [45]. Of note, about 10–15% HPV-positive HNSCC patients have an activating mutation in the coding region of the *PIK3CA* gene, making it the most common mutation [90]. HPV-positive OPC has the highest number of *PIK3CA* mutations compared with other HPV-negative tumours [106]. PI3K is regulated by tumour suppressor phosphatase and tensin homolog (*PTEN*). Lui’s et al. discovered *PTEN* gene copy loss in 4/45 HNSCC cases [90], and this can be seen in both HPV-positive and HPV-negative tumours [76, 124].

Mutations in *HRAS* gene are seen in low frequencies (5%) in both HPV-positive and HPV-negative HNSCC [139]. *HRAS* is an oncoprotein, which interacts with the PI3K complex in a GTP-dependent manner to increase the catalytic activity of PI3K kinase [125].

Although the NOTCH pathway is oncogenic in some types of cancer, its role in HNSCC seems to be tumour suppressive [117, 139]. Nearly 66% of HNSCC tumours carry genetic mutation in at least one member of the NOTCH pathway [3]. An in vitro study indicated that abrogated or absent *NOTCH1* causes loss of proliferation and senescence in HNSCC cell lines [117]. Approximately 15% of patients with HNSCC (both HPV-negative and HPV-positive) have *NOTCH1* mutation [3, 117, 139].

*FBXW7* is a member of F-box protein family and acts as a tumour suppressor gene that mainly targets NOTCH1 [3]. Its mutations are mostly seen in HPV-negative HNSCC, with little proportion in HPV-positive [134]. Lechner et al. reported copy number variations of *FBXW7* in HPV-positive HNC [76], whilst Agrawal et al. reported indels and missense mutations [3]. It is hypothesized that mutation of *FBXW7* can modulate the NOTCH pathway. Studies have also shown that HNSCC patients with *TP53* mutations had significantly higher mutation rates in *FBXW7* [108].

E1A binding protein P300 (*EP300)* is located on chromosome 22 and acts as a histone acetyltransferase. It regulates transcription by chromatin remodeling [30]. It is also involved in the NOTCH pathway, which affects cell growth and apoptosis.

cAMP-response element binding protein-BP (*CREBBP*) acts as a tumour suppressor gene and encodes a protein that participates in chromatin remodeling. It is reported to have loss-of-function mutations in many types of malignancies [87] and closely related to paralogue *EP300*. Loss of function of *CREBBP/EP300* is documented to increase the proliferation ability of tumour cells [51].

*KMT2D* is a tumour suppressor gene encoding histone-lysine N-methyltransferase 2D, which is vital for embryonic development, and it is widely expressed in adult tissue. Mutation in *KMT2D* are common in a number of cancers, HNC, brain, bladder, prostate, and lung [40]. Frameshift and nonsense mutations in the SET and PHD domains represent 37% and 60% respectively of *KMT2D* total mutations [119]. Mutation in *KMT2D* can affect H3K4me1-marked enhancer [15] regulation, which is a possible mechanism leading to cancer development. In addition, genomic instability during DNA replication and transcription can cause abnormalities in early replicating fragile sites in the chromosome, leading to DNA breaks and formation of tumour [66]. Furthermore, *MAPK1* (Mitogen-Activated Protein Kinase 1) mutation (p.D321N and E322K) correlates to Erlotinib sensitivity in HNC patients [105, 164].

### cfDNA isolation and detection technologies

cfDNA is separated either by using centrifugal columns or magnetic beads [93] for downstream applications. ctDNA represents only a very small percentage of the total cfDNA, making it very challenging when isolating and detecting it. Based on the analysis, ctDNA technologies can be divided into three categories, single locus or multi-loci, targeted sequencing and whole genome sequencing (WGS) (Table 1). Single loci or multiplex assays, with a rapid turnaround time, are applied mainly to detect/quantify hotspot mutations and to monitor recurrent mutations [159]. For the detection of multi-loci mutations, PCR amplicons and hybrid-capture assays are commonly used [42]. While amplicon-based sequencing has better “on-target” effects, hybridization capture has higher uniformity [126]. In general, hybridization capture method requires > 1 μg DNA (SeqCap is an exception), but amplicon-based sequencing requires only 10–100 ng of total DNA. Non-targeted sequencing can detect unknown genomic alterations, such as detecting chromosomal structural variants by using WGS [159].

**Table 1:** Comparison of ctDNA mutation analysis technologies [159]

Generation	Scale of analysis	Examples of technologies	Applications	Advantages	Disadvantages
First generation and next generation sequencing	Single locus or limited multiplex assays	PCR-based: **· **Droplet Digital PCR (ddPCR) [140] **· **BEAMING [31] **· **Intplex [149]	**· **Detection and quantification of selected loci, cancer hotspot mutations and small number of mutations	**· **Widely used **· **High sensitivity	**· **Sampling error can happen when samples need to split into multiple reactions **· **Very low copy numbers of mutant DNA impairs the overall performance **· **Only detects previously known mutations
		Enrichment for mutant alleles: **· **COLD-PCR [82] **· **SCODA [95] **· **NaME-PRO [136]			
Next generation sequencing	Targeted sequencing assays	Amplicon-based method: **· **SiMSen-Seq [144] **· **TAM-Seq [38] **·** Enhanced TAM-Seq [41] **·** Safe-SeqS [71] ·AmpliSeq [143] ·MiSeq [69] ·NextSeq [67] ·HaloPlex [83]	**· **Genotyping **· **Interrogating numerous mutations (SNP, CNV, insertion, deletion, structural variation) **·**Individual exons of interest to the whole exome	**· **Reduced background error rates because of higher coverage compared to WGS **· **Detection of allele fractions below 0.1% **· **Even with limited amounts of input samples, sensitivity can be further enhanced	· Only detecting mutations in targeted genes/locations · Limited detection of fusions
		Hybridization capture: **· **CAPP-Seq [104] **· **TARDIS [97] **· **SureSelect [74] **· **SeqCap [26] **· **HiSeq [81]			
	Non-targeted assays or Genome-wide	Whole genome sequencing (WGS): **· **Plasma-Seq [58] **· **PARE [75]	**· **Identification of amplifications and deletions **· **Detection of fetal aneuploidies	**· **Investigation of genomic alterations without previously knowledge **· **Characterization of the whole molecular landscape	**· **Limited detection in low tumour purity samples (< 25%) allele fractions sensitivity poor below low coverage **· **Limited sensitivity for profiling early-stage cancer **· **High cost
		Amplicon-based: **· **FAST-SeqS [70] **· **mFast-SeqS [9]			
Third generation sequencing	Targeted and whole genome sequencing	**·** Oxford Nanopore Technology (ONT) **·** CyclomicsSeq [92]	**·**Detection of a wide range of variants from SNV to structural variants **·** Methylation of cfDNA	**· **Rapid turnaround time **· **Flexible accessibility **· **Cost-effective **· **Unrestricted read length (20 bp to 4 Mb)	**·** Relatively high error rates

Abbreviations used in Table 1 (in alphabetical order)

AmpliSeq: sequencing amplified, BEAMING: beads, emulsion, amplification, magnetics, CAPP-Seq: cancer personalized profiling by deep sequencing, COLD-PCR: co-amplification at lower denaturation temperature, FAST-SeqS: fast aneuploidy screening test-sequencing system, HaloPlex: The Agilent HaloPlex Target Enrichment System, mFAST-SeqS: modified fast aneuploidy screening test-sequencing system, NaME-PRO: nuclease-assisted minor-allele enrichment with probe-overlap, PARE: personalized analysis of rearranged ends, Safe-SeqS: safe-sequencing system, SCODA: synchronous coefficient of drag alteration, SiMSen-Seq: Simple, multiplexed, PCR-based barcoding of DNA for sensitive mutation detection using sequencing, TAM-Seq: tagged amplicon deep sequencing, TARDIS: targeted digital sequencing

Besides the first generation and the next-generation sequencing technologies, Oxford Nanopore Technology (ONT) is the third-generation sequencing that relies on the detection of electrical changes as nucleic acids passing through a protein nanopore. This technology is predominantly used in sequencing long-length sequences, such as genomic DNA [163]. More recently, Marcozzi et al. [92], developed a new technique based on the ONT, CyclomicsSeq, which is able to detect ctDNA *TP53* mutation at frequencies down to 0.02%. Details of ctDNA detection technologies are summarized in Table 1.

### Applications of ctDNA

**Fig. 3:**
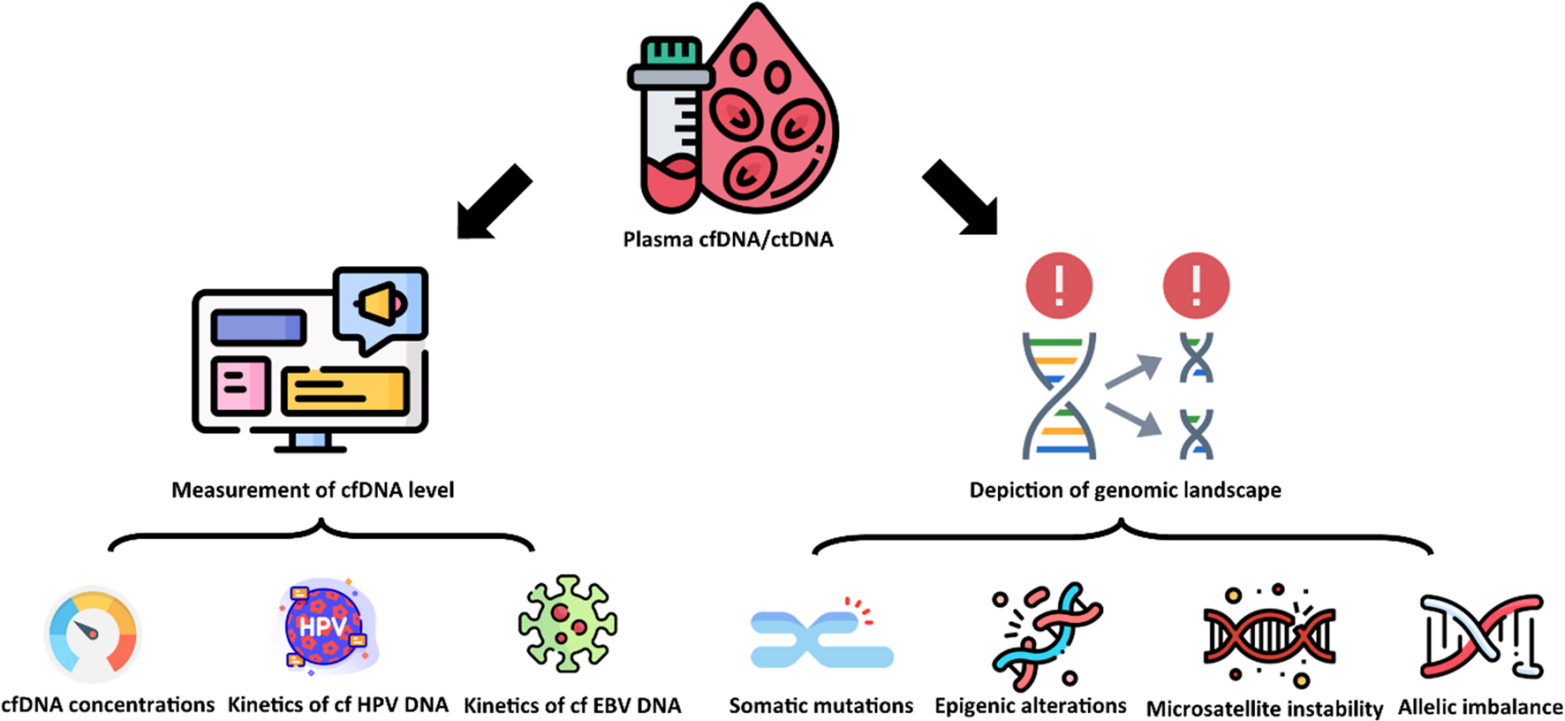
Application of cfDNA/ctDNA (Icons made by Flaticon, www.flaticon.com). cfDNA can be used to quantified levels and depict cancer genomic landscape

ctDNA has been widely applied in the early detection of cancer, predict tumour burden, monitor response to treatment [129]. As illustrating in Fig. 3, researchers have used a wide range of tumour specific markers to capture tumour activity using ctDNA (Fig. 3).

### Quantification of cfDNA levels

Levels of cfDNA is associated with the stage of the tumour and can indicate disease progress. Hilke et al. sequenced 20 tumour samples from locally advanced HNSCC patients and followed them longitudinally during and post treatment and found that 85% of patients had detectable cfDNA and that the amount of cfDNA correlated with the gross tumour volume [59]. Lin et al. analyzed plasma samples from 121 patients with OSCC and concluded that a higher level of plasma cfDNA were related to a poor prognosis, indicating that cfDNA levels could serve as a prognostic biomarker [86]. Egyud et al. reported that 50% of HNSCC patients (N = 4) had detectable cfDNA levels prior to recurrence, indicating that cfDNA can be applied as a biomarker to early detect recurrence [34]. Mazurek et al. detected lower levels of cfDNA in HNSCC patients (N = 200) compared with cfDNA levels detected in OPSCC patients. HNSCC patients with late stage (T2, T3 and stage IV) tumours had higher cfDNA levels than those patients from early stages of the disease [96], which is not surprising. In addition, Burgener et al. reported shorter fragment lengths of cfDNA from HNSCC patients (N = 30) compared to healthy controls (N = 20). In contrast, Shukla et al. reported no significant differences in cfDNA levels between OSCC patients (N = 390) and a control group (N = 150) [135]. Furthermore, HNSCC patients who had detectable cfDNA at baseline (collection of blood at diagnosis) were more likely to develop advanced disease and as a consequence showed poorer overall survival [13].

### Biomarkers captured on ctDNA

#### HPV viral DNA

Circulating HPV DNA (ctHPVDNA) has widely been used as a biomarker in disease prediction and treatment monitoring in patients with HNSCC. Cao et al. reported that pre-treatment ctHPVDNA copy number was closely associated with the metabolic activity of lymph nodes and tumour volume in a 64 HNC patient cohort. A reduction in ctHPVDNA copy number was seen in 14 patients receiving chemoradiotherapy. Similarly, the ctHPVDNA levels were elevated in 13 HNSCC patients coinciding with the time of metastasis, further providing evidence that ctHPVDNA levels can be used as a prognostic biomarker [17]. Dahlstrom et al. reported in 262 patients with OPSCC, pre-treatment ctHPVDNA levels were associated with a higher dissemination of cancer cells to lymph nodes, increasing the overall disease stage. HPV-positive OPSCC patients showed better progression-free survival than HPV-negative patients [27]. Hanna et al. discovered that the plasma ctHPVDNA levels were associated with tumour burden and metastatic potential in 22 OPSCC patients. In addition, they also showed that the copy number of ctHPVDNA levels increased in patients with metastasis. They concluded that levels of ctHPVDNA was linked to treatment response and corelated with survival [54]. A separate study by the same group compared the ctHPVDNA levels in paired saliva and plasma samples from OPSCC patients (N = 21) and revealed that ctHPVDNA levels in both fluids can be used as a biomarker of disease surveillance [53]. Damerla et al. reported that 90/97 patients with OPSCC had detectable ctHPVDNA and ctHPV16DNA in 100% of patients with low-volume disease (N1 or an isolated T1-2). Also, the copy numbers of ctHPV16DNA levels reduced after surgery and/or chemoradiation [28]. Chera et al. in 2019 reported that pre-treatment ctHPV16DNA in 103 OPSCC patients were linked to tumour burden. In addition, a rapid clearance profile of HPV DNA may predict disease control [22]. A more recent longitudinal study by the same group in 2020 reported that in 87 patients with undetectable ctHPVDNA at all the post-treatment time points, none of them had developed recurrence (NPV, 100%; 95% CI, 96–100%) [23]. Only 28 patients had detectable ctHPVDNA levels during post-treatment surveillance, 15 of them were diagnosed with recurrence which was proved by tissue biopsy. 15/16 patients who were detected to have two consecutively positive ctHPVDNA blood tests had developed biopsy-proven recurrence. Two consecutively positive blood test of ctHPVDNA indicated a positive predictive value of 94% (95% CI, 70–99%). The median lead time between positivity of ctHPVDNA and recurrence proven by tissue biopsy was 3.9 months (range, 0.37–12.9 months) [23]. Similarly, Reder et al. concluded that elevated ctHPVDNA levels were associated with tumour size based on a study involving 50 OPSCC patients. Whilst OPSCC patients with continuously high levels of ctHPVDNA developed residual disease or recurrence (5/8), patients without recurrence had decreased ctHPVDNA after treatment (N = 25) [121]. In a mono-institutional prospective biomarker study by Veyer et al. using OPSCC patients (p16-positive/HPV16-positive) reported 47 patients (71%) showed ctHPVDNA at the time of diagnosis. Moreover, the abundance of baseline ctHPV16DNA levels being assessed by ddPCR, was significantly related to the T/N/M status and tumour stages. Furthermore, all recurrences and the majority of death (83%) were reported to have positive baseline ctHPV16DNA. The kinetic of pretreatment or posttreatment ctHPVDNA (N = 6) was apparently co-related to treatment success or failure [158]. Haring et al. reported ctHPV16DNA test in HPV-positive recurrence/metastasis OPSCC patients (N = 16) could predict progressive disease prior to radiographic imaging [55]. Rettig et al. [123], reported ctHPVDNA also had pre-diagnostic value, since they could detect HPV16 several years before the onset of HPV16-related HNSCC. Among 10 patients diagnosed with HPV16-positive tumour, three of them were found to had ctHPVDNA at least six months before the diagnosis.

However, it is worth mentioning that even though the above studies all focused on ctHPVDNA, they used different probes of HPV, controls, and analysis methods. The following Table 2 summarized the probes or analysis method in each study.

**Table 2:** An overview analysis method in ctHPVDNA studies

Study	Detection regions	Analysis method	Control
Cao et al. [17]	HPV L1, HPV 16/18, E6, E7	TaqMan PCR	β-globin
Dahlstrom et al. [27]	HPV16 E6, E7	Real-time PCR	β-actin
Hanna et al. [54]	HPV16,18,31,33,45 E7	Droplet digital PCR	pUC57 plasmid
Hanna et al. [53]	HPV16,18,31,33,45 E7	Multiplexed droplet digital PCR	pUC57 plasmid
Damerla et al. [28]	HPV16, HPV33	Droplet digital PCR	*EIF2C1*
Chera et al. [22]	HPV16,18,31,33,45 E7	Droplet digital PCR	*ESR1*
Chera et al. [23]	HPV16 E6, 37; HPV18,31,33,35,37 E7	Digital PCR	*ESR1*
Reder et al. [121]	HPV16 E6, E7	Real-time quantitative PCR	β-globin
Veyer et al. [158]	HPV16 E6	Droplet digital PCR	Albumin gene
Haring et al. [55]	HPV16 E6	TaqMan probe-based ddPCR	UM-SCC-104, UM-SCC-105
Retting et al. [123]	HPV16, 18, 31, 33, 35	Droplet digital PCR	*ESR1*

#### EBV viral DNA

Infection with Epstein-Barr Virus (EBV) contributes to the development of nasopharyngeal carcinoma (NPC) [21]. Similar to ctHPVDNA, circulating plasma EBV (ctEBVDNA) or cell-free EBV DNA (cfEBVDNA) [20, 78] have been used as a prognostic biomarker for investigating tumour burden, treatment response and disease progression [21]. He et al. reported that the present of ctEBVDNA in 949 NPC patients at multiple time points of treatment was associated with poor overall survival (OS), distant metastasis free survival (DMFS), and progression-free survival (PFS) [57]. Lin et al. reported higher concentrations of ctEBVDNA in NPC (N = 99) patients who relapsed than those who did not. Furthermore, NPC patients with persistently detectable ctEBVDNA had shorter OS than those with undetectable ctEBVDNA [85]. Similarly, Edward et al. reported a rapid decrease in ctEBVDNA levels post-surgery in 21 NPC patients. Importantly, they documented that failure of rapid elimination of ctEBVDNA was predictive for disease recurrence [151]. Chen et al. conducted a longitudinal study involving 1984 NPC patients and found that during the follow-up, 767/1984 NCP patients had detectable ctEBVDNA, and among them, 489/767 (63.8%) developed recurrence. Thus, they concluded that ctEBVDNA can be an early indicator of tumour recurrence [20].

### Mutations

ctDNA mutation profiles have been evaluated to monitor response to treatment in HNSCC patients [129, 130]. However, the application of such technology is still in its infancy. The current clinical practice is to profile tumour tissue for mutation and then track these mutations using ctDNA. This works well when the concentration of ctDNA levels is high, as seen in patients with metastatic cancer. However, this approach fails when ctDNA amounts are low, which is the case for most non-metastatic cancers. To overcome this issue, a study by Burgener et al. combined both mutation and methylation analysis and found that 20 out of 30 HNSCC patients had similar mutation frequencies to that of the tumour data derived from TCGA data base [13]. In addition, there was a correlation (r > 0.85) between mutations and methylation levels. HNSCC patients who had detectable pre-treatment ctDNA (using mutation and methylation) showed worse overall survival (HR = 7.5; P = 0.025) independent of clinical stages. Schirmer et al. compared copy number aberrations (CNAs) and genome-wide copy number instability score (CNI) and showed that the CNI may assist in predicting lymph node involvement and prognosis in HNSCC [128].

Schwaederle et al. analyzed ctDNA from various cancer types (HNC = 25) and concluded that HNC was an independent predictor for a higher number of alterations in ctDNA (P = 0.019, median of 3 alterations (95%CI 1–68%) [133]. Braig et al. found that over one third of HNSCC patients showed acquired *KRAS, NRAS* or *HRAS* mutations after cetuximab treatment [12]. van Gink et al. reported *TP53* mutations in plasma of six HPV-negative HNSCC patients [156]. *PIK3CA* E545K mutations were detected in the plasma samples from 9/29 HNSCC patients [129].

### Tumour and ctDNA mutations

When compared with other cancer types, there is dearth of data relating to tumour tissue mutations and ctDNA mutation profiling in HNSCC. Table 3 highlights studies that have used both tumour and ctDNA. This further infers that cfDNA can be used as a proximity to tumour DNA to determine outcomes in patients. More so, ctDNA holds unique mutation profiles, thus giving clinicians the opportunity to early detect minimal residual disease and may also provide new insights for therapy choice.

**Table 3:** Selected studies comparing HNSCC mutation profiles between tumour DNA (tDNA) and ctDNA

Author and publish year	Patient cohort and sample types	Methods	Results and conclusions
Perdomo et al. 2017 [115]	-Targeted mutation gene panel: fresh tumour tissue and plasma ctDNA from 36 HPV-negative HNSCC patients -Non-targeted approach using TP53 gene mutation: fresh tumour tissue, plasma ctDNA and oral rinse samples from 37 HNSCC patients	-Targeted approach: using a European cohort of patients, a 5-gene mutation panel (*TP53, PTEN, NOTCH1, CASP8* and *CDKN2A*) -Non-targeted approach: the whole coding region of *TP53* gene using a South American cohort of patients	-Targeted approach: 42% (15/36) of cases had detectable plasma ctDNA mutations, 67% were from early stages (I, II) cases. 18 mutations were detected in both plasma ctDNA the matched tDNA with allelic fractions (AF) ranging from 0.001 to 0.12 -Nontargeted: One case showed (*TP53* Arg174Trp) exclusively in plasma ctDNA Four cases showed concordance in *TP53* mutation between tumour tissue and oral rinse samples One case showed concordance in *TP53* mutation among tumour tissue, plasma, and oral rinse
Porter et al. 2019 [118]	Tumour specimen (N = 30) and plasma ctDNA (N = 60) from patients with recurrent and metastatic HNC	-Tumour DNA: Foundation One and Caris -ctDNA: Guardant Health Inc	-*TP53* (48% vs 68%) and *PIK3CA* (24% vs 34%) were two most common mutations in tDNA and ctDNA -Patients who had ctDNA and tDNA sequencing (N = 30), 20/30 had alterations in tDNA and ctDNA ctDNA identified a new mutation at 73% (22/30)
Mes et al. 2020 [99]	Frozen tumour tissue and matching plasma ctDNA from 27 HNSCC patients	-Illumina HiSeq -Illumina MiSeq	-Concordance rate of copy number variation (CNV) between tDNA and ctDNA was 52% (14/27) -Some mutations were only identified in ctDNA but not in matching tDNA. (*CASP8, NSD1, KMT2D, CDKN2A, NOTCH1, TP53*)
Wilson et al. 2020 [165]	Tumour tissue samples and plasma ctDNA from 75 HNSCC patients	-Foundation One Platform (323 genes) tDNA -Guardan360 platform in ctDNA	-13% concordance between tDNA and plasma ctDNA *-TP53* was the most concordant gene -*BRCA1, EGFR, KIT, BRAF, ESR1, FGFR2, FGFR3, MAP2K1* and *NRAS* shared similar alteration in both tDNA and ctDNA -*ARID1A, ATM and MET* showed more alteration in ctDNA than in tDNA -65.3% of patients had detectable actionable alteration in ctDNA
Galot et al. 2020 [43]	Plasma from 39 recurrent/metastatic HNSCC patients, and matched tumour FFPE from 18 HNSCC patients	-A 604 gene mutation panel -ddPCR	- The most frequently mutated gene in ctDNA was *TP53*, followed by genes in the PI3K pathway -Compared to locoregional recurrent disease, a higher probability of detecting ctDNA was found in HNSCC patients in metastatic disease (70% versus 30%) -81% of mutations identified in solid tumours were not detected in ctDNA -26% of ctDNA variants were not detected in matched tDNA - The cancer driver events identified in both tDNA and matching ctDNA were *TP53, MYC, EGFR, CDKN2A* and *PIK3CA*
Khandelwal et al. 2020 [69]	Frozen tumour tissue and plasma samples from 22 OPSCC patients	MiSeq platform	- 12/22 mutations were identified in tDNA· - 11/22 mutations were identified in plasma cfDNA· - HPV-negative OPSCC non-responder patients were more likely to have variant detected in ctDNA -Five patients carried six matching mutations (*TP53* G325fs, *TP53* R282W, *TP53* R273C, *FBXW7* R505G, *FBXW7* R505L, *TP53* Q331H) in both tDNA and ctDNA - A high concordance was found between *TP53* mutation in ctDNA and tumour tissue in HPV-negative OPSCC
Burgener et al. 2021 [13]	Plasma ctDNA from 30 HPV-negative HNSCC patients; and TCGA data- derived tumour mutational information	CAPP-seq for ctDNA mutation analysis	-Common cancer driver mutations in *TP53, PIK3CA, FAT1* and *NOTCH1* were found between tDNA and ctDNA - Mutations in two genes (*GRIN3A* and *MYC*) were only specific to ctDNA
Liebs et al. 2021 [83]	HNSCC patients (N = 6) tumour tissue FFPE and matching plasma ctDNA samples	A targeted 327 cancer gene panel; The HaloPlex™ HS target enrichment system	-A relatively low (11%) mutation concordance rate was found between tDNA and ctDNA -With high input of ctDNA(> 30 ng), five genes (*ACACA, ATR, LAMA2, PIK3CA* and *SMARCA4*) were mutated both in tDNA and ctDNA - With low input DNA (< 30 ng), 52 mutations in 3 genes (*FAT1, RELN* and *PDGFRA*) were found in tDNA -With 30 ng input of tDNA and ctDNA, a total of nine mutations in four genes were found in cfDNA, (*EZH2, LAMA2, PIK3CA* and *SMARCA4*) -Analyzed ctDNA followed by their correspondent tDNA with low input (< 30 ng), 2/6 mutations (*EPHA2* and *FLT3*) existed in both types of DNAs
Kogo et al. 2022 [72]	Frozen tumour tissue and plasma from 26 HNSCC patients	-A targeted gene panel comprising 31 genes for tDNA; -ddPCR for ctDNA	The most frequently mutated gene is *TP53* (58.2%). Longitudinal positivity of ctDNA revealed prognosis
Flach et al. 2022 [37]	Tumour FFPE and plasma from HNSCC patients (N = 8)	Oncomine. Comprehensive Assay	*TP53* was the most frequently mutated gene. 37.5% variants were shared between tDNA and ctDNA

### DNA methylation

When regulatory regions of tumour suppressor genes are methylated (tumour suppressor genes), their expression levels are reduced, leading to the development of tumour [84, 110]. Sanchez et al. investigated the methylation alterations in common tumour suppressor genes (*CDKN2A*, *MGMT, GSTP1, and DAPK1*) in primary tumour samples and matched serum samples from HNSCC patients (N = 50). They found similar DNA methylation profiles between tumour tissue and serum DNA (21 and 50 respectively). For those patients with serum positive hypermethylated DNA, 5 out of 21 developed recurrence, while only 1 out of 29 patients who relapsed had negative serum methylation DNA [127]. Tian et al. analyzed the promoter hypermethylation of five tumour suppressor genes in blood samples from NPC patients (N = 41) and healthy controls (N = 41). They reported percentage methylations of *RASSF1* (17.5%), *CDKN2A* (22.5%), *DLEC1* (25.0%), *DAPK1* (51.4%) and *UCHL1* (64.9%). When combining four-gene methylation markers (*CDKN2A, DLEC1, DAPK1* and *UCHL1*) in predicting NPC, it gave the highest sensitivity and specificity [150]. Mydlarz et al. detected *EDNRB*, *p16* and *DCC* methylation by analyzing serum DNA from HNSCC patients and revealed that serum *EDNRB* hypermethylation was highly specific for HNSCC but it was not sensitive [102]. Schröck et al. showed that methylation levels of *SHOX2* and *SEPT9* in serum from HNSCC patients (N = 284) correlated with tumour and nodal category and was associated with higher risk of death [131]. Jesus et al. compared methylation status of *CCNA1*, *DAPK, CDH8* and *TIMP3* between FFPE tumour samples (N = 52) and corresponding plasma samples (N = 15). They detected methylation in 73% of plasma samples, while methylation of *CCNA1* was related to recurrence-free survival [29]. Patel et al. [112], compared methylation profiles of pre-treatment and post-treatment ctDNA in HNC patients (N = 8). Significant methylation changes have been seen in the promoter regions of *PENK, NXPH1, ZIK1, TBXT* and *CDO1* between pre-treatment and post-treatment ctDNA. Table 4 highlights genes that are mutated and methylated in HNSCC.

**Table 4:** Mutated and methylated genes in HNSCC ctDNA

Mutated genes in ctDNA	Studies
*TP53*	Burgener et al*.* [13]; Flach et al. [37]; Galot et al. [43]; Khandelwal et al. [69]; Kogo et al. [72]; Mes et al. [99]; Perdomo et al. [115]; Porter et al. [118]; van Gink et al. [156]; Wilson et al. [165]
*PIK3CA*	Burgener et al. [13]; Galot et al. [43]; Liebs et al. [83]; Porter et al. [118]*,*
*MYC*	Burgener et al. [13]; Galot et al. [43]*,*
*CDKN2A*	Galot et al. [43]; Mes et al. [99]*,*
*NRAS*	Braig *et.al.,* [12]; Wilson et al. [165]*,*
*EGFR*	Galot et al. [43]; Wilson et al. [165]*,*
*NOTCH1*	Burgener et al. [13]; Mes et al. [99]*,*
*FAT1*	Burgener et al. [13]; Liebs et al. [83]*,*
*BRCA1, KIT, BRAF, ESR1, FGFR2, FGFR3, MAP2K1, ARID1A, ATM, MET*	Wilson et al. [165]*,*
*ACACA, ATR, LAMA2, SMARCA4, RELN, PDGFRA, EZH2*	Liebs et al. [83]*,*
*CASP8, NSD1, KMT2D,*	Mes et al. [99]*,*
*KRAS, HRAS*	Braig *et.al.* [12]*,*
*GRIN3A*	Burgener et al. [13]*,*
*FBXW7*	Khandelwal et al. [69]*,*
Methylated genes in ctDNA	
* CDKN2A*	Sanchez et al. [127]; Tian et al. [150]*,*
* DAPK1*	Sanchez et al. [127]; Tian et al. [150]*,*
* CCNA1, DAPK, CDH8, TIMP3*	Jesus et al. [29]*,*
* PENK, NXPH1, ZIK1, TBXT, CDO1*	Patel et al. [112]*,*
* EDNRB, p16, DCC*	Mydlarz et al. [102]*,*
* RASSF1, DLEC1, UCHL1*	Tian et al. [150]*,*
* MGMT, GSTP1*	Sanchez et al. [127]*,*
* SHOX2, SEPT9*	Schröck et al. [132]*,*

### Microsatellite instability

Microsatellite sequences are short non-coding repeat sequences that vary in length between individuals. Nawroz-Danish et al. reported that 45% (68/152) of HNSCC patients had microsatellite alterations in the DNA isolated from serum samples and was identical to the alterations observed in corresponding tumour samples. Furthermore, 16 HNSCC patients with distant metastasis, 11 had detectable microsatellite alterations in DNA derived from serum with one or more markers [103]. Nunes et al. compared microsatellite alterations in 91 paired blood and tumour samples from HNSCC patients and found that 58 of the tumour tissues displayed microsatellite alterations, 29% also exhibited the same alterations in ctDNA [107]. Kakimoto’s et al. discovered that 90% of OSCC patients (N = 20) showed microsatellite alterations in serum DNA, with 10 patients showing allelic imbalance post-operative serum DNA. 70% patients showed an allelic imbalance at pre-operation and post-operation, with a poor prognosis [65].

### Allelic imbalance

Allelic imbalance (AI) is a condition in which the expression levels of two alleles of the same gene differ in the same cell, either as a result of the epigenetic inactivation of one of the alleles or as a result of genetic changes in the regulatory regions. Hamana et al. analyzed AI in tumour tissue and serum samples from OSCC patients (N = 64) at three time points (pre-operatively, post-operatively, and 4 weeks post-operatively) and found that 52% patients’ serum samples showed AIs in at least one locus and AIs were frequently detected pre-operatively and post-operatively. Importantly, OSCC patients who had AIs during the post-operative period but turned negative 4 weeks post-operatively were free of disease. In contrast, patients who were AI-positive both post-operatively and 4 weeks post-operatively deceased due to distant metastasis. Therefore, microsatellite status in the serum DNA could be used as a potential biomarker in monitoring disease progression [52]. Jiang et al. analyzed plasma DNA length (integrity index) in HNSCC patients (N = 58) and control subjects (N = 47) and concluded that plasma DNA integrity index was increased in HNCC patients compared with non-cancerous healthy controls [63].

### Combining biomarkers present in blood and saliva samples as a means of increasing cancer detection rates

Ahn et al. investigated HPV16DNA levels in saliva and plasma samples from OPSCC patients (N = 93) pre-treatment and post-treatment. For pre-treatment samples combining saliva and serum, the sensitivity, specificity, negative predictive value, and positive predictive values of HPV16DNA were 76%, 100%, 42%, and 100% respectively [4]. Similarly, Wang et al. analyzed saliva and plasma samples from 93 HNSCC patients and reported that tumour DNA (referred to either somatic mutations or human papillomavirus genes) detection rate was 100% in early-stage patients, 95% in late-stage disease, 100% in OC, 91% in OPC, and 100% in LC. In saliva, tumour DNA was detected in all the patients with OC and 70% of patients with cancers from other sites. In plasma, tumour DNA was detected in 80% of patients with OC, and all the patients with cancers from other sites [162]. Hanna et al. discovered that paired blood-saliva HPV DNA can be used in disease surveillance [53]. Carvalho et al. analyzed salivary oral rinse, serum and tumour tissues from 211 HNSCC patients and 527 healthy controls. They used quantitative methylation specific PCR as well as a 21-gene panel and concluded that compared to single marker, combining data from saliva and serum samples showed an improved detection [18].

### Future perspective

Next-generation sequencing (NGS) of tumour tissue DNA is emerging as a promising avenue to comprehensively characterize tumour mutation burden. High nonsynonymous tumor mutational burden (TMB), evaluated by WES has shown to correlate with improved clinical outcomes for patients with other types of cancer (lung cancer). However, the use of tumour biopsies to discern clinically available biomarkers have limitations. These include tumour heterogeneity, access to tumour tissues in anatomically challenging locations, insufficient quantity of tumour DNA and the inability to monitor response to treatment in patients who have undergone surgical resections.

Liquid biopsy-based applications are revolutionizing the management of patients with cancer [73]. Studies have shown that using NGS to capture tumour specific mutations is an emerging field of importance to track response to treatment. To confirm whether ctDNA recapitulates de novo tumour tissue genomic landscape, increasing studies are comparing tumour tissue DNA from a patient with HNSCC to their ctDNA derived from blood. As an example, when a drug targets a particular mutation, analysing whether ctDNA carries the same mutation, would allow more precise delivery of treatment, enabling a precision medicine approach. We envisioned that in the future ctDNA analysis will become part of routine clinical management of HNSCC patients, whereby improving outcomes through targeted therapies.

## Conclusion

The lack of biomarkers to triage the risk of relapse at diagnosis, disease surveillance and predicting recurrence are considered as the main contributors for poor outcomes in HNSCC. To date, most of the research in liquid biopsies has focused on blood-based biomarkers, predominantly using ctDNA. The analysis of ctDNA has several benefits over traditional tumour biopsy testing. Liquid biopsy enables real-time monitoring of response to treatment, also including those patients with tumours in anatomically challenging locations. However, well-designed multi-center clinical trials using homogeneous HNSCC patient cohorts where the use of ctDNA as a biomarker for disease management should provide meaningful benefits to patients before it is broadly implemented clinically.

## Data Availability

Not applicable.
